# Serum zinc concentration and extracellular water/total body water ratio on admission may be independent predictors of the prognosis of patients with terminal colorectal cancer

**DOI:** 10.20407/fmj.2026-007

**Published:** 2026-09-25

**Authors:** Akihiro Ito, Masanobu Usui, Miyo Murai, Norimasa Tsuzuki, Akihiko Futamura, Kazuki Imai

**Affiliations:** Department of Surgery and Palliative Medicine, Fujita Health University, School of Medicine, Tsu, Mie, Japan

**Keywords:** Terminal colorectal cancer, Prognostic factor, Serum zinc, Extracellular water, Total body water

## Abstract

**Objectives:**

In palliative care, it is important to accurately predict the prognosis of patients with cancer. Therefore, we aimed to identify prognostic factors for patients with terminal colorectal cancer.

**Methods:**

Of the 756 patients with terminal cancer admitted for palliative care who subsequently died over a 3-year period starting in April 2018, 59 (7.8%) had colorectal cancer, did not die within 7 days of admission, and were studied. At admission, the circulating concentrations of cachexia-related proteins, tumor markers, and essential trace elements were measured. Additionally, body composition was analyzed by bioelectrical impedance analysis using an InBody S10. Prognostic factors were identified using multivariate analysis.

**Results:**

The median age of the patients was 80 years (26 male and 33 female). The median duration of survival from admission was 35 days (interquartile range: 20–55 days). After adjustment for potential confounding factors (age, sex, the Eastern Cooperative Oncology Group performance status, and body mass index), multivariate analysis revealed that the serum zinc concentration (hazard ratio [HR]: 0.9804; 95% confidence interval [CI]: 0.9658–0.9938; *p*=0.0066) and the extracellular water/total body water (ECW/TBW) ratio (HR: 1.3587; 95% CI: 1.1062–1.6753; *p*=0.0037) were independent prognostic factors. The optimal cutoff value of ECW/TBW to categorize 4-week survival was calculated to be 0.421.

**Conclusions:**

The serum Zn concentration and the ECW/TBW ratio were shown to be independent predictors of the prognosis of patients with terminal colorectal cancer at the commencement of palliative care.

## Introduction

In palliative care for patients with terminal cancer, the accurate prediction of prognosis is important for the maintenance of quality of life (QOL) until the end of life and to facilitate the provision of high-quality medical care that reflects the wishes of patients and their families. The incidence of colorectal cancer is increasing in Japan, owing to the Westernization of dietary habits. According to the 2024 Vital Statistics Summary of the Ministry of Health, Labour, and Welfare in Japan, the annual number of deaths from cancer was 384,099. Colorectal cancer ranks as the second leading cause of cancer-related mortality overall, following lung cancer, but it is the leading cause among women, and the number of deaths from this form is increasing annually.^1^

The prevalence of cancer cachexia varies with the type of cancer, with colorectal cancer being among the most frequent causes.^2^ Patients with cachexia are characterized by a severe systemic inflammatory response and a reduction in physical activity, which lead to a decline in QOL.^3^ Therefore, predicting the prognosis of patients with terminal colorectal cancer is crucial.

There have been a number of studies of potential prognostic factors for use in palliative care. Murai et al. reported that the interleukin (IL)-8 concentration and clinical symptoms are prognostic factors for patients with advanced cancer and cachexia. They also noted that those with a shorter prognosis had significantly lower serum albumin (Alb) and transthyretin (TTR) concentrations and significantly higher C-reactive protein (CRP) concentrations than those with a longer prognosis.^4^ Miura et al. demonstrated that the Glasgow Prognostic Score, which uses the serum Alb and CRP concentrations, is a useful predictor of prognosis for patients with cancer undergoing palliative care.^5,6^ They also reported that the serum TTR concentration is a useful nutritional index and prognostic factor in this setting.^7^ Furthermore, we previously reported that the serum concentrations of essential trace elements, such as zinc, significantly correlate with the concentrations of these cachexia-related proteins in patients with terminal cancer.^8^ However, to date, there have been no studies in which specific prognostic factors for terminal colorectal cancer were identified.

Patients with terminal colorectal cancer often develop ascites, owing to carcinomatous peritonitis, liver dysfunction, secondary to multiple liver metastases, or edema, resulting from malnutrition, anemia, and hypoalbuminemia. Because of this, the use of body composition analysis through bioelectrical impedance analysis (BIA) has recently been gaining attention. BIA enables the measurement of muscle mass to assess sarcopenia and the extracellular water/total body water (ECW/TBW) ratio to assess edema. However, although their values are expected to be abnormal in patients with terminal colorectal cancer, there have been no studies of the potential use of BIA-derived data as prognostic factors in this specific population. Therefore, in the present study, we aimed to characterize the relationships between prognosis and various parameters (the serum concentrations of cachexia-related proteins, tumor markers, and essential trace elements, and BIA-derived body composition parameters) in patients with terminal colorectal cancer.

## Methods

### Study sample and definitions

Of the 756 patients with terminal cancer who were admitted to Fujita Health University Nanakuri Memorial Hospital for palliative care between April 2018 and March 2021 and died during hospitalization, 59 with colorectal cancer (7.8%) were included in the present study, after excluding those who died within 7 days of admission. In this study, “terminal cancer” was defined by multiple physicians at a review meeting on the basis of objective clinical data, such as those obtained by diagnostic imaging and laboratory testing, showing that the disease was incurable and that the predicted survival time was approximately 3 months or less. The study was approved by the Medical Research Ethics Review Committee of Fujita Health University (authorization no. HM16-401, approval date : January 12. 2018).

### Laboratory assessments

Routine blood tests were performed upon admission. These included measurements of the serum concentrations of cachexia-related markers (transthyretin [TTR], albumin [Alb], and C-reactive protein [CRP]), tumor markers (carcinoembryonic antigen [CEA] and carbohydrate antigen 19-9 [CA19-9]), and essential trace elements (zinc [Zn], iron [Fe], and copper [Cu]). Blood samples were collected under fasting conditions, before breakfast, and the same serum samples were used for all the assays. The reference values for the serum TTR, Alb, CRP, CEA, and CA19-9 concentrations were 22–44 mg/dL, 4.0–5.0 g/dL, <0.7 mg/dL, <5.0 ng/mL, and <37 U/mL, respectively. For the trace elements, the reference ranges were 65–135 μg/dL for Zn, 65–200 μg/dL (men) and 50–160 μg/dL (women) for Fe, and 68–128 μg/dL for Cu.

### Body composition analysis

Body composition was evaluated within 2 days of admission using an InBody S10^®^ (InBody, Tokyo, Japan) BIA device. We assessed the skeletal muscle index (SMI) as an indicator of sarcopenia, using cutoff values of <7.0 kg/m^2^ for men and <5.7 kg/m^2^ for women.^9^ It was calculated as each patient’s appendicular skeletal muscle mass divided by the square of their height (kg/m^2^). Additionally, the ECW/TBW ratio was used as an index of edema, and the phase angle (PhA) was measured as an index of cell membrane integrity.

### Statistical analysis

Continuous data sets are presented as medians with interquartile ranges (IQRs). Univariate analysis (Wald test) was used to identify factors significantly associated with survival (*p*<0.05). Multivariate analysis was then performed using the Cox proportional hazards model (Wald test) and the parameters that were significant in the univariable analysis to identify independent prognostic factors, after adjustment for potential confounding factors (age, sex, the Eastern Cooperative Oncology Group performance status [ECOG PS], and body mass index [BMI]). For each identified independent prognostic factor, the patients were divided into two groups on the basis of the median value, and survival curves were created using the Kaplan–Meier method and compared using the log-rank test. The median values were used as cutoff values for the Kaplan–Meier analysis because clinical reference ranges were not applicable: nearly all the patients had values of these parameters outside the normal ranges upon admission. These values usefully stratified the severity of the physiological impairment of the sample. Furthermore, cutoff values for 4-week (approximately 1-month) survival—a clinically significant milestone for patients with terminal cancer and their families—were established, and the areas under the receiver operating characteristic curves (AUCs) and the associated *p*-values were calculated. We also evaluated the scatter plot and Spearman’s rank correlation coefficient for the relationship between the serum zinc and cachexia-associated protein (TTR, Alb, and CRP) concentrations. All the statistical analyses were performed using JMP Pro version 18.0 software (SAS Institute Inc., Cary, NC, USA).

## Results

### Patient characteristics and laboratory data

The median age of the patients was 80 years; 26 were male and 33 were female. The tumors were located in the right colon in 26 patients, the left colon in 16, and the rectum in 17. The median duration of survival from the date of admission was 35 days (IQR: 20–55 days). At the time of admission, the patients had abnormal cachexia-related marker concentrations: the median serum TTR was 9.3 mg/dL (IQR: 6.6–13.0 mg/dL), and 57 patients (96.6%) had values below the reference range; and the median Alb concentration was 2.6 g/dL (IQR: 2.2–3.0 g/dL), and 58 patients (98.3%) had values below the reference range. Conversely, the median CRP concentration was 4.8 mg/dL (IQR: 2.0–8.6 mg/dL), and 54 patients (91.5%) had values above the reference range. Regarding tumor markers, there were high median serum concentrations of CEA (248 ng/mL; IQR: 26.0–1,130.4 mg/dL) and CA19-9 (237.4 U/mL; IQR: 39.7–2,117.9 U/mL). The analysis of essential trace elements revealed that the median serum Zn concentration was 55 μg/dL (IQR: 47–64 μg/dL), and 45 patients (76.3%) had values below the reference range. The median serum Fe concentration was 28 μg/dL (IQR: 14.5–38 μg/dL), and 56 patients (94.9%) had values below the reference range. Finally, the median serum Cu concentration was 127 μg/dL (IQR: 89–153 μg/dL); six patients (10.2%) had values below the reference range, and 29 (49.2%) had values above this.

### Body composition of the patients

The body composition analysis indicated low muscle mass; the median SMI for the male participants was 4.82 kg/m^2^ (IQR: 4.16–6.35 kg/m^2^), and for the female participants it was 3.41 kg/m^2^ (IQR: 2.94–4.02 kg/m^2^). Notably, all the patients were diagnosed with sarcopenia on the basis of sex-specific criteria (<7.0 kg/m^2^ for the men and <5.7 kg/m^2^ for the women). The median ECW/TBW, an indicator of edema, was high, at 0.423 (IQR: 0.414–0.434), and 94.9% of the patients had an ECW/TBW ratio ≥0.40. The median PhA was low, at 2.7° (IQR: 2.1–3.3°) (Table 1).

### Predictors of prognosis and survival

The relationships between prognostic indices and survival time are summarized in Table 2A. Univariate analysis showed that the serum CRP (*p*=0.0181) and Zn (*p*=0.0066) concentrations and the ECW/TBW (*p*=0.0037) were significantly associated with survival. The indices that showed no significant association in univariate analysis (the serum TTR, Alb, Fe, and Cu concentrations, SMI, and PhA) remained non-significant in the multivariate analysis.

Multivariate analysis using the Cox proportional hazards model, after adjustment for potential confounding factors (age, sex, ECOG PS , and BMI), identified the serum Zn concentration (hazard ratio [HR]: 0.9804; 95% confidence interval [CI]: 0.9658–0.9938; *p*=0.0066) and ECW/TBW (HR: 1.3587; 95% CI: 1.1062–1.6753; *p*=0.0037) as independent predictors of prognosis (Table 2B).

Kaplan–Meier survival analysis revealed that patients with an ECW/TBW ≥0.421 (the median value) had significantly shorter survival times than those with values <0.421 (*p*=0.0280). Similarly, patients with serum Zn concentrations <58 μg/dL (the median value) had significantly shorter survival times than those with values ≥58 μg/dL (*p*=0.0185) (Figure 1).

### Predictive performance for 4-week survival

We also evaluated the cutoff values for 4-week survival, a significant milestone. For ECW/TBW, the cutoff value was 0.421, with an AUC of 0.69079 (*p*=0.0125). For the serum Zn concentration, the cutoff value was 57.0 μg/dL, but the AUC was lower (0.5994) and the result was not statistically significant (*p*=0.0772) (Figure 2).

## Discussion

Cancer cachexia in patients with terminal disease is characterized by a significant loss of skeletal muscle and adipose tissue.^10,11^ Pathophysiologically, it is defined by negative protein and energy balance, resulting from poor oral intake and metabolic abnormalities.^12^ Previous studies have shown that systemic markers of inflammation, such as CRP and the CRP-to-albumin ratio, are predictors of the prognosis of patients with colorectal cancer and cachexia.^13^ In the present study, we evaluated the relationships between survival and cachexia-related proteins (the serum TTR, Alb, and CRP concentrations). Univariate analysis indicated that only serum CRP was a significant predictor of the prognosis of patients with terminal colorectal cancer. Shrotriya et al. reported in a systematic review that CRP is a biomarker of the prognosis of recurrent solid tumors.^14^

As tumors grow, invade surrounding tissues, and metastasize, the resulting tissue destruction and inflammatory response trigger CRP production. High CRP concentrations suggest more severe tissue inflammation and destruction, which are associated with a poorer prognosis. Furthermore, the serum CRP concentration correlates with that of IL-6, which reflects tumor proliferative activity and malignancy.^15^ Therefore, we hypothesized that CRP would be an independent prognostic factor in multivariate analysis. However, it was not significant, which may be explained by CRP being an acute phase reactant that shows sharp increases in concentration during infections such as pneumonia. In the present cohort, 10 patients (16.9%) exhibited clinical signs of acute bacterial infection (fever [temperature ≥38°C] and leukocytosis [≥10,000 leukocytosis/mm^3^]). Owing to the nature of palliative care, many patients declined intensive diagnostic imaging (e.g., computed tomography), making it difficult to exclude the impact of concurrent infections on their CRP concentrations.

Regarding body composition, BIA is a particularly useful technique, because it is rapid, non-invasive, and reproducible. It permits the tracking of changes in body composition, such as muscle mass and edema, and the reliability of BIA-derived measurements has been validated in multiple studies. Modern multi-frequency BIA devices, such as the InBody S10 used in the present study, use high- and low-frequency currents to accurately distinguish ECW from ICW, without the assumption of constant tissue hydration. Previous research has demonstrated that BIA measurements are highly reproducible, provided that consistent posture and lead placement are maintained.^16^ Although we expected the SMI to correlate with prognosis because it reflects advancing cachexia, no significant correlation was found. As we previously suggested following a study of pleurodesis, this may be because edema within muscle tissue is misidentified as muscle mass by BIA, resulting in an overestimation of SMI in patients with terminal cancer.^17^ Additionally, although the PhA reflects cell membrane integrity and functional status, it was not found to be an independent prognostic factor, probably because it is influenced by multiple factors, including age and overall physique. In contrast, the ECW/TBW ratio—an excellent indicator of edema—was an independent predictor of prognosis.

In healthy individuals, the ECW/TBW ratio is approximately 0.38, and values exceeding 0.40 indicate overhydration.^18^ In the present study, 94.9% of the patients met this criterion. High ECW/TBW ratios in patients with terminal colorectal cancer reflect a complex interplay of malnutrition, hypoalbuminemia, ascites secondary to carcinomatous peritonitis, and hepatic or renal dysfunction. Furthermore, systemic inflammation increases vascular permeability, promoting the shift of fluid to the extracellular space. It is important to note that a high ECW/TBW ratio in patients with terminal cancer reflects not only simple fluid overload, but also a more profound, global physiological decline. From a cellular perspective, as catabolism progresses in cancer cachexia, the integrity of cell membranes diminishes, leading to a reduction in intracellular water content. Simultaneously, systemic inflammation compromises sodium/potassium pump function, causing an imbalance in which water shifts from the intracellular to the extracellular compartment. Therefore, the ECW/TBW ratio should be interpreted as a holistic marker that reflects nutritional depletion, inflammatory activity, and cellular frailty. The present findings are consistent with those of Zheng et al., who identified ECW/TBW ≥0.40 as an indicator of poor prognosis in patients with advanced cancer,^19^ but extend its utility to patients with terminal colorectal cancer specifically.

The physiological mechanism linking a high ECW/TBW ratio to poor prognosis is likely to be complex. Chronic systemic inflammation involves the release of proinflammatory cytokines, which increase vascular endothelial permeability, facilitating the leakage of fluid into the interstitial space. Concurrently, the metabolic demands of the tumor and worsening cachexia lead to severe hypoalbuminemia, further reducing the plasma oncotic pressure and exacerbating the edema. These processes signify a state of irreversible metabolic collapse and multi-organ dysfunction, which is consistent with the shorter survival of patients in the present study.

Our previous study of patients with terminal cancer demonstrated that the circulating concentrations of essential trace elements significantly correlate with those of cachexia-related proteins such as TTR, Alb, and CRP.^8^ Specifically, the serum Zn concentration significantly correlated with the serum TTR (r=0.4531, *p*<0.0001), Alb (r=0.4882, *p*<0.0001), and CRP (r=–0.4168, *p*<0.0001) concentrations. However, there have been no studies of terminal colorectal cancer specifically or of the relationship between the serum Zn and prognosis of these patients. The present study has revealed that serum Zn is an independent predictor of the prognosis of patients with terminal colorectal cancer. Heyland et al. reported that in patients with cancer, the concentrations of inflammatory cytokines such as IL-6 and TNF-α increase as part of chronic inflammation and cachexia, and promote hepatic zinc uptake.^20^ This phenomenon is a defensive mechanism, but it further reduces the circulating serum Zn concentration.

Persistent inflammation and stress may also alter the expression of zinc transporters, affecting their intra- and extracellular distribution and prolonging systemic zinc deficiency. Additionally, Wo et al. conducted a prospective study of patients with colorectal cancer, and found that those with Zn concentrations <85.6 μg/dL had a significantly higher incidence of distant metastasis and lower survival rates than those with concentrations above this threshold.^21^ Patients with terminal cancer are at particular risk of Zn deficiency, owing to the following factors: (1) inadequate oral intake, (2) malabsorption because of impaired intestinal function or malabsorption syndrome, (3) greater excretion or loss through the gastrointestinal tract or urine, and (4) interactions with other trace elements.^22^

In the present study, although TTR and Alb, which are closely related to malnutrition and cachexia, were not found to be independent prognostic factors, the serum Zn concentration significantly positively correlated with both the TTR (r=0.4718, *p*=0.0002) and Alb (r=0.3646, *p*=0.0045) concentrations. In terminal colorectal cancer, the Alb and TTR concentrations are often low, thereby limiting their utility in the determination of a prognosis. In contrast, the serum Zn concentration may not only reflect protein/energy malnutrition, but also the functional exhaustion of the body’s trace element reservoirs, providing a more granular assessment of the “point of no return” in metabolic collapse.

A significant inverse correlation between the serum Zn and CRP concentrations (r=–0.4168 was identified in our previous study).^8^ During chronic inflammation associated with cancer cachexia, Zn is redistributed from the intravascular space to the liver and other tissues as part of the acute phase response. Therefore, a low serum Zn concentration in such patients may reflect both nutrient depletion and the intensity of the underlying inflammatory process that drives cancer progression. In the present study, there were 10 patients (16.9%) in whom fever was present at the time of admission and leukocytosis was present, suggesting the likelihood of concomitant acute bacterial infection. There was no correlation between the serum CRP and Zn concentrations (r=–0.1949, *p*=0.1390). These findings suggest that serum Zn represents a robust predictor of prognosis in patients with terminal colorectal cancer, reflecting the progressive decline in nutritional status and worsening malabsorption. The present results suggest that serum Zn concentration may be a more sensitive and objective indicator of the progression of nutritional and metabolic collapse in patients with terminal colorectal cancer than conventional protein markers.

The potential clinical utility of measuring the serum Zn concentration lies in its objectivity and accessibility. In palliative care, during which invasive testing is often minimized, a single baseline Zn measurement upon admission could provide clinicians with a powerful tool for prognostic stratification. Specifically, it could assist with the identification of patients who may no longer benefit from aggressive nutritional interventions, but instead require a shift in focus toward comfort-oriented care and psychological support for their families. However, the cutoff value (57.0 μg/dL) for 4-week survival—a significant clinical milestone—was associated with a small AUC of 0.5994, and the serum Zn concentration was not statistically significant. This indicates that whereas serum Zn was independently associated with overall survival time in the multivariate analysis, its performance as a binary predictive tool for a specific time point was suboptimal.

Based on the above results, we will consider future clinical applications. The multivariable analysis of data from the patients at the time of admission indicated that the serum Zn concentration and the ECW/TBW ratio are independent prognostic factors. For prognosis assessment at the time of admission, the probability of 4-week survival could be categorized using an ECW/TBW ratio of approximately 0.421.

The ECW/TBW cutoff value identified exceeds the standard edema threshold (0.40). A ratio of 0.421 may indicate significant fluid redistribution owing to systemic inflammation and hypoalbuminemia. Clinically, this may be an indicator for the commencement of fluid restriction to prevent worsening edema-related distress, as recommended in palliative care guidelines. Although the serum zinc cutoff value (57.0 μg/dL) had limited predictive accuracy (a small AUC) for 4-week mortality specifically, this value may represent a threshold for severe metabolic wasting. Clinically, values below this cutoff suggest that the patients are entering a stage of refractory cachexia, and therefore it may be time to shift the focus of management from nutritional restoration to symptom palliation. Moreover, because the serum Zn concentration was an independent predictor of prognosis, we can speculate that maintaining nutritional status after admission is important to support treatment plans.

The nutritional status of patients with cachexia deteriorates for multiple reasons, and therefore a multifaceted approach is probably necessary. In addition to increasing nutritional intake using oral nutritional supplementation (ONS), nutritional counselling, physical exercise therapy, and the appropriate management of adverse symptoms have been recommended for implementation in conjunction with treatment of the underlying disease.^23^ Furthermore, many patients with advanced cancer cachexia become resistant to nutritional therapy because of anabolic resistance. Therefore, it is important to start nutritional therapy before the nutritional status deteriorates, to minimize avoidable problems that worsen nutritional status, such as inadequate nutritional intake (starvation) and a lack of exercise, maintain nutritional status, and prevent the progression of cachexia.^23^

ONS has also been reported to be a first-line nutritional therapy to increase oral nutrient intake.^23,24^ The nutrition support teams at Nanakuri Memorial Hospital, Fujita Health University, are actively working to improve patient nutrition. However, the number of patients who can undergo ONS administration is limited, and many cannot receive it within a few days. The effectiveness of home parenteral nutrition has also been reported for patients who survive for more than 3 months under conditions of starvation and malnutrition.^25^ In the present study, body composition analysis revealed a median ECW/TBW ratio, an indicator of fluid retention (edema), of 0.423 (IQR, 0.414–0.434).

An ECW/TBW ratio of ≥0.40 is used to diagnose edema. In Japan, the Clinical Guidelines for Infusion Therapy in Patients with Advanced Cancer recommends limiting fluid intake to <1,000 mL/day in patients with terminal cancer and a life expectancy of approximately 1 month who have edema, to alleviate the associated pain.^26^ Nanakuri Memorial Hospital also follows this guideline and has a policy of not using excessive fluid therapy. However, it has been suggested that in the future, for patients with an ECW/TBW ratio of <0.40, active parenteral nutrition may be considered as a means of maintaining and improving nutritional status. For this reason, nutritional management using parenteral nutrition was excluded from consideration in the present study. However, nutritional management during hospitalization (oral intake, enteral nutrition, and parenteral nutrition) is considered to be extremely important, and its omission represents a limitation of this study.

Other limitations of this study include the following. (1) It was conducted in a single institution. Therefore, the outcomes of the study may not apply to other institutions. (2) We did not categorize the causes of death into chronic cancer progression *versus* acute events, such as perforation or hemorrhage. (3) The various nutritional support modalities (oral *vs.* parenteral) were not compared. (4) Pharmacological interventions, such as antibiotics and corticosteroids, post-admission were not accounted for. (5) Although the locations of the primary tumor were identified, a detailed analysis of the metastatic sites, such as liver metastasis and peritoneal dissemination, was not performed because of incomplete records. (6) The effects of prior chemotherapy were not fully accounted for. Because these clinical factors can directly influence systemic inflammatory responses, trace element metabolism, and the overall severity of cachexia, their exclusion might have influenced the prognostic outcomes. These factors should be addressed in future research.

In conclusion, in patients with terminal colorectal cancer, the serum TTR, Alb, and Zn concentrations frequently indicated malnutrition, and the CRP concentration reflected a state of chronic inflammation. Body composition analysis showed that 94.9% of the patients had edema (ECW/TBW ≥0.40). Multivariate analysis identified the serum Zn concentration and the ECW/TBW ratio as independent prognostic factors, and therefore these may represent valuable objective metrics for end-of-life clinical assessments. In particular, the optimal cutoff value of ECW/TBW for 4-week survival was calculated to be 0.421.

## Conflicts of interest

The authors declare no conflicts of interest associated with this research.

## Figures and Tables

**Figure 1:**
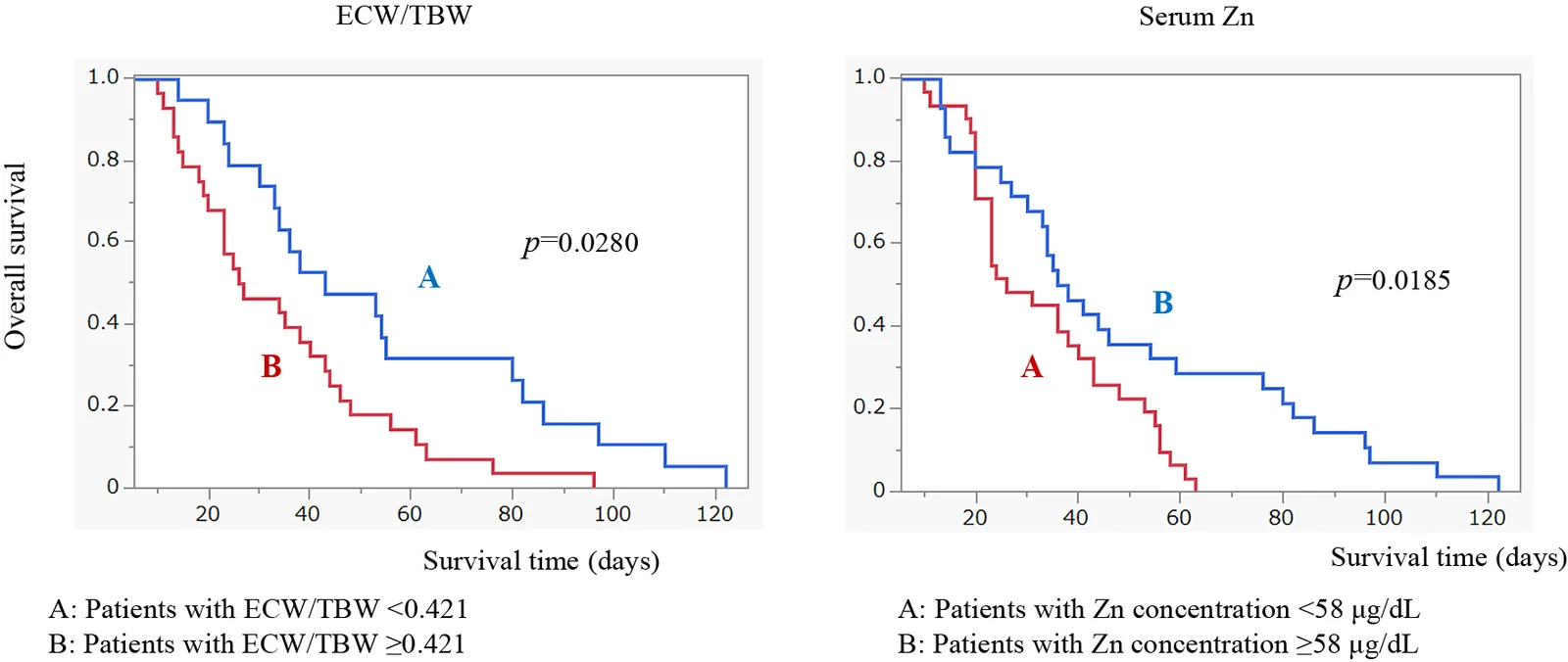
Survival times of the patients, categorized according to each median value

**Figure 2:**
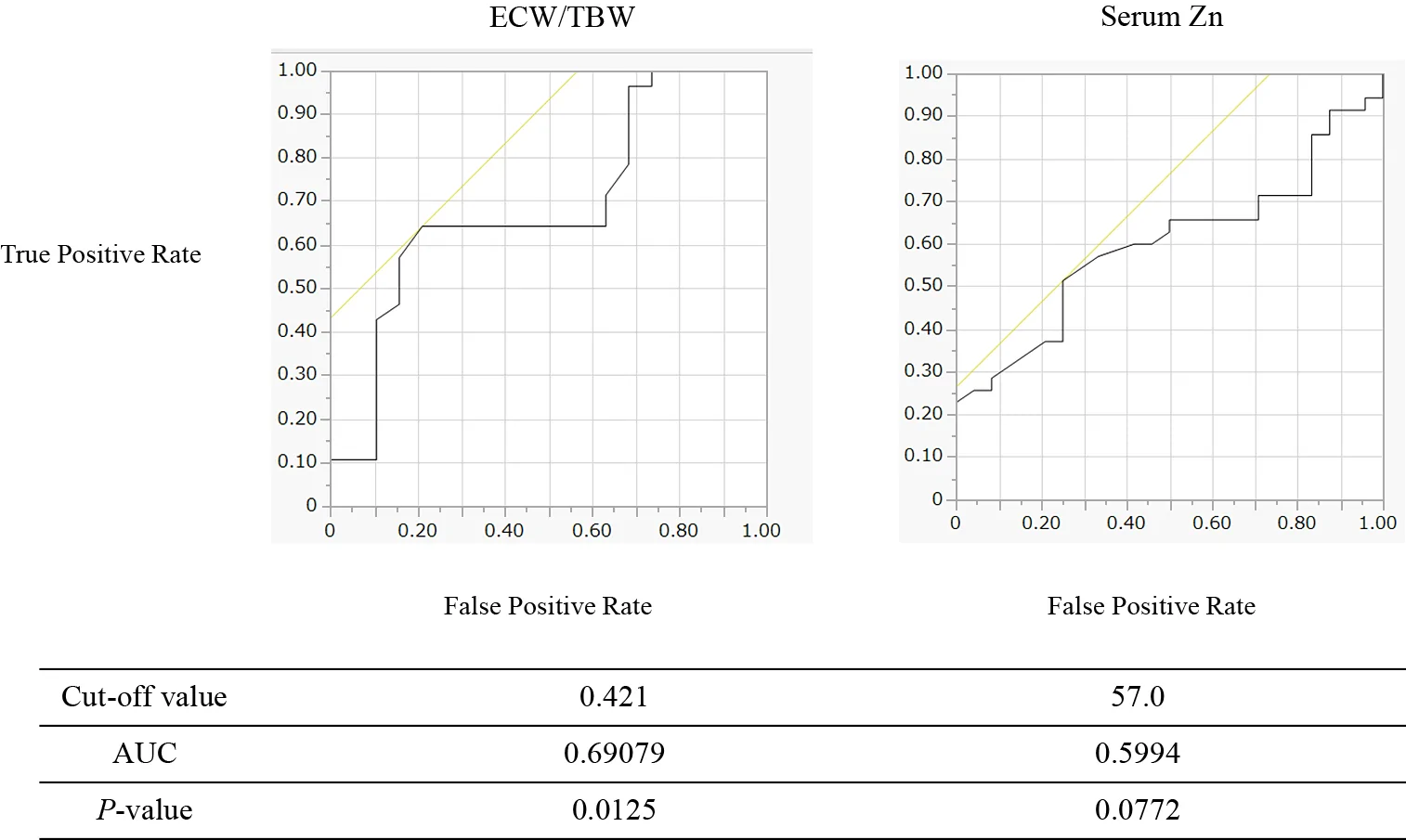
Determinations of each cut-off value using the ROC curve for 4-week survival AUC, area under the receiver operating characteristic curve

**Table 1:** Characteristics of the patients

Parameter	n=59
Age, years, median (IQR)	80 (44–96)
Sex, n (%)	59
male	26 (44.1)
female	33 (55.9)
Body mass index, kg/m^2^, median (IQR)	20.3 (17.3–22.4)
ECOG PS, n (%)	
1	0 (0)
2	22 (37.3)
3	27 (45.8)
4	10 (16.9)
Tumor location, n (%)	
right colon	26 (44.1)
left colon	16 (27.1)
rectum	17 (28.8)
Laboratory data	
Transthyretin, mg/dL, median (IQR)	9.3 (6.6–13.0)
Albumin, g/dL, median (IQR)	2.6 (2.2–3.0)
C-reactive protein, mg/dL, median (IQR)	4.8 (2.0–8.6)
Carcinoembryonic antigen, ng/mL, median (IQR)	248 (26.0–1130.4)
Carbohydrate antigen 19-9, U/mL, median (IQR)	237.4 (39.7–2117.9)
Serum Zn, μg/dL, median (IQR)	55 (47–64)
Serum Fe, μg/dL, median (IQR)	28 (14.5–38)
Serum Cu, μg/dL, median (IQR)	127 (89–153)
Body composition analysis	
Skeletal muscle mass index, kg/m^2^, median (IQR)	
Male	4.82 (4.16–6.35)
Female	3.41 (2.94–4.02)
ECW/TBW, median (IQR)	0.423 (0.414–0.434)
Phase angle, °, median (IQR)	2.7 (2.1–3.3)
Hospital stay, days, median (IQR)	35 (20–55)

ECOG PS: Eastern Cooperative Oncology Group performance status. ECW/TBW: extracellular water/total body water. IQR: interquartile range.

**Table 2A:** Results of the univariable Cox regression analyses for mortality, based on laboratory data collected at admission

	Univariable analysis for prognosis (days of survival)		
	Hazard ratio	95% CI	*P*-value
Age	0.9982	0.9708–1.0280	0.9046
Sex			0.7530
male/female	0.9576	0.6460–1.8298	
female/male	0.9198	0.5465–1.5481	
ECOG PS	1.5689	1.1060–2.2073	0.0103
Body mass index	1.0558	0.9858–1.1278	0.1126
Serum transthyretin	0.9576	0.9069–1.0030	0.0909
Serum albumin	0.7959	0.4800–1.3149	0.3737
Serum C-reactive protein	1.0696	1.0093–1.1289	0.0181
Serum carcinoembryonic antigen	1.0001	1.0000–1.0002	0.0537
Serum carbohydrate antigen 19-9	1.0000	1.0000–1.0000	0.4806
Serum Zn	0.9804	0.9658–0.9938	0.0066
Serum Fe	0.9947	0.9857–1.0013	0.1735
Serum Cu	1.0003	0.9942–1.0066	0.9356
Skeletal muscle mass index			
Male	1.3402	0.9867–1.8057	0.0525
Female	1.3975	0.8381–2.2730	0.1838
ECW/TBW×100%	1.3587	1.1062–1.6753	0.0037
Phase angle	0.8377	0.5804–1.1536	0.3094

ECOG PS: Eastern Cooperative Oncology Group performance status. CI: confidence interval.

**Table 2B:** Results of the univariable and multivariable Cox regression analyses for mortality, based on laboratory data collected at admission

Parameter	Univariable analysis				Multivariable analysis※										
					For C-reactive protein				For ECW/TBW				For Serum Zn		
	HR	95% CI	*P*-value		HR	95% CI	*P*-value		HR	95% CI	*P*-value		HR	95% CI	*P*-value
Age	0.9982	0.9708–1.0280	0.9046		0.9768	0.9458–1.0106	0.1651		0.9871	0.9541–1.0238	0.4688		0.9780	0.9474–1.0119	0.1861
Sex			0.7530				0.7554				0.7285				0.9019
male/female	1.0871	0.6495–1.8298			0.9141	0.5196–1.0681			1.1224	0.5848–2.1542			1.0359	0.5912–1.8149	
female/male	0.9198	0.5465–1.5481			1.0939	0.6218–1.9244			0.8910	0.4642–1.7100			0.9654	0.5510–1.6913	
ECOG PS	1.5689	1.1060–2.2073	0.0103		1.6213	1.0996–2.3514	0.0123		1.3511	0.8038–2.1781	0.2335		1.5761	1.0516–2.3173	0.0234
Body mass index	1.0558	0.9859–1.1278	0.1126		1.0326	0.9529–1.1143	0.4199		1.0502	0.9521–1.1538	0.3146		1.0334	0.9550–1.1133	0.3997
C-reactive protein	1.0696	1.0093–1.1289	0.0181		1.0572	0.9891–1.1230	0.0841								
ECW/TBW	1.3587	1.1062–1.6753	0.0037						1.2693	1.0185–1.5805	0.0324				
Serum Zn	0.9804	0.9658–0.9938	0.0066										0.9832	0.9679–0.9972	0.0252

※ after adjustment for age, sex, performance status, and body mass index. HR: Hazard Ratio. CI: confidence interval.
